# Supplementary material for: Productivity, nutrient use efficiency, energetic, and economics of winter maize in south India
Source: PLoS One. 2022 Jul 21;17(7):e0266886. doi: 10.1371/journal.pone.0266886 (PMC9302768; doi:10.1371/journal.pone.0266886)
Supplement: S1 File — (DOCX) [file pone.0266886.s001.docx]

**S1 Table. Physico-chemical properties of soil in the experimental site**

| **Sl. No.** | **Particulars** | **Value** | **Method adopted** |
| --- | --- | --- | --- |
| **I. Physical properties** | | | |
| a. | Particle size analysis |  | International pipette method (Piper, 2002) |
|  | Coarse sand (%) | 6.23 |  |
|  | Fine sand (%) | 12.66 |  |
|  | Silt (%) | 28.17 |  |
|  | Clay (%) | 52.93 |  |
|  | Textural class | Clay |  |
| **II. Chemical properties** | | | |
| a. | Soil pH (1:2.5 soil: water suspension) | 7.6 (Neutral) | Buckman’s pH meter (Piper, 2002) |
| b. | Electrical conductivity  (1:2.5 soil: water suspension) (dS m^-1^) | 0.33 | Conductivity bridge (Piper, 2002) |
| c. | Organic carbon (%) | 0.45 | Walkley and Buck wet oxidation method (Jackson, 1973) |
| d. | Available nitrogen (kg ha^-1^) | 261  (Low) | Modified alkaline potassium permanganate method (Sharawat and Burford, 1982) |
| e. | Available phosphorus (kg ha^-1^) | 31.5  (Medium) | Olsen’s method (Sparks, 1996) |
| f. | Available potassium (kg ha^-1^) | 289  (Medium) | Flame photometry method (Sparks, 1996) |

**S2 Table. Monthly meteorological data during 2019-20 and the average of 69 years (1950-2018) at the Main Agricultural Research Station, Dharwad**

| **Month** | **Rainfall (mm)** | | **Rainy days** | **Mean temperature (^o^C)** | | | | **Relative humidity (%)** | |
| --- | --- | --- | --- | --- | --- | --- | --- | --- | --- |
|  | **1950-2018** | **2019-20** | **2019-20** | **Maximum** | | **Minimum** | | **1950-2018** | **2019-20** |
|  |  |  |  | **1950-2018** | **2019-20** | **1950-2018** | **2019-20** |  |  |
| April-19 | 38.8 | 54.5 | 6 | 36.5 | 37.2 | 20.2 | 21.0 | 58.6 | 53.0 |
| May-19 | 78.1 | 16.2 | 1 | 35.2 | 37.2 | 21.2 | 21.5 | 64.3 | 56.9 |
| June-19 | 109.4 | 104.7 | 6 | 29.6 | 31.5 | 21.1 | 21.5 | 79.7 | 76.0 |
| July-19 | 136.3 | 230.8 | 17 | 27.2 | 27.1 | 20.7 | 20.3 | 86.3 | 87.4 |
| August-19 | 104.6 | 451.2 | 17 | 26.8 | 26.4 | 20.4 | 20.4 | 86.1 | 87.6 |
| Sept-19 | 103.8 | 106.8 | 10 | 28.4 | 27.3 | 20.0 | 20.2 | 81.7 | 80.1 |
| October-19 | 106.4 | 323.2 | 12 | 29.7 | 27.9 | 19.0 | 19.0 | 75.1 | 79.8 |
| Nov-19 | 32.0 | 21.0 | 2 | 29.7 | 29.7 | 18.1 | 18.1 | 69.5 | 68.9 |
| Dec-19 | 8.5 | 7.8 | 1 | 28.8 | 28.6 | 16.5 | 16.5 | 67.3 | 69.4 |
| January-20 | 0.8 | 0.0 | 0 | 28.9 | 29.8 | 14.6 | 15.5 | 53.2 | 60.5 |
| February-20 | 10.7 | 0.0 | 0 | 31.7 | 31.8 | 16.1 | 16.8 | 44.9 | 49.4 |
| March-20 | 5.7 | 13.6 | 2 | 34.9 | 34.0 | 18.6 | 19.4 | 42.5 | 49.0 |
| **Total/Mean** | **735.1** | **1329.8** | **74** | **30.6** | **30.7** | **18.6** | **20.9** | **67.4** | **69.0** |

**S3 Table. Number of green leaves per plant at different growth stages of *rabi* maize as influenced by sowing windows and fertility levels**

| **Treatment** | **Number of green leaves per plant** | | |
| --- | --- | --- | --- |
|  | **30 DAS** | **60 DAS** | **90 DAS** |
| **Factor I: Sowing windows** | | | |
| W_1_: 1^st^ week of October | 8.8^a^ | 13.3^a^ | 11.4^a^ |
| W_2_: 2^nd^ week of October | 8.6^a^ | 13.2^a^ | 11.0^ab^ |
| W_3_: 3^rd^ week of October | 7.9^b^ | 12.4^ab^ | 10.4^bc^ |
| W_4_: 4^th^ week of October | 7.0^c^ | 11.5^bc^ | 10.3^c^ |
| W_5_: 1^st^ week of November | 6.5^c^ | 11.0^c^ | 9.6^d^ |
| **S.Em ±** | **0.17** | **0.27** | **0.18** |
| **Factor II: Fertility levels** | | | |
| F_1_: 100 % RDF | 7.5^b^ | 11.7^b^ | 10.3^a^ |
| F_2_: 150% RDF | 7.7^ab^ | 12.4^ab^ | 10.5^a^ |
| F_3_: 200% RDF | 8.1^a^ | 12.7^a^ | 10.8^a^ |
| **S.Em ±** | **0.21** | **0.31** | **0.23** |
| **Interaction (W×F)** | | | |
| W_1_F_1_: 1^st^ week of Oct + 100 % RDF | 8.5^a-c^ | 12.5^b-e^ | 11.3^a^ |
| W_1_F_2_: 1^st^ week of Oct + 150 % RDF | 8.7^ab^ | 13.5^a-e^ | 11.4^a^ |
| W_1_F_3_: 1^st^ week of Oct + 200 % RDF | 9.1^a^ | 13.9^a^ | 11.5^a^ |
| W_2_F_1_: 2^nd^ week of Oct + 100 % RDF | 8.5^a-c^ | 12.7^a-d^ | 10.6^a-c^ |
| W_2_F_2_: 2^nd^ week of Oct + 150% RDF | 8.3^a-c^ | 13.3^a-c^ | 11.1^ab^ |
| W_2_F_3_: 2^nd^ week of Oct + 200 % RDF | 9.1^a^ | 13.8^a^ | 11.4^a^ |
| W_3_F_1_: 3^rd^ week of Oct + 100 % RDF | 7.8^b-e^ | 12.2^a-e^ | 10.3^a-c^ |
| W_3_F_2_: 3^rd^ week of Oct + 150 % RDF | 7.9^a-e^ | 12.4^a-e^ | 10.4^a-c^ |
| W_3_F_3_: 3^rd^ week of Oct + 200 % RDF | 8.1^a-d^ | 12.5^a-e^ | 10.5^a-c^ |
| W_4_F_1_: 4^th^ week of Oct + 100 % RDF | 6.7^ef^ | 10.7^de^ | 10.1^a-c^ |
| W_4_F_2_: 4^th^ week of Oct + 150 % RDF | 7.1^d-f^ | 11.6^b-e^ | 10.3^a-c^ |
| W_4_F_3_: 4^th^ week of Oct + 200 % RDF | 7.3^c-f^ | 12.1^a-e^ | 10.6^a-c^ |
| W_5_F_1_: 1^st^ week of Nov + 100 % RDF | 6.3^f^ | 10.5^e^ | 9.4^c^ |
| W_5_F_2_: 1^st^ week of Nov + 150 % RDF | 6.5^f^ | 11.1^de^ | 9.5^c^ |
| W_5_F_3_: 1^st^ week of Nov + 200 % RDF | 6.8^ef^ | 11.3^c-e^ | 9.8^bc^ |
| **S.Em ±** | **0.37** | **0.59** | **0.40** |
| C_1_: Control | 5.0 | 7.9 | 8.5 |
| **S.Em ±** | **0.39** | **0.59** | **0.39** |

**S4 Table. Leaf area and leaf area index at different stages of *rabi* maize as influenced by sowing windows and fertility levels**

| **Treatment** | **Leaf area (dm^2^ plant^-1^)** | | | | | **Leaf area index** | | | |
| --- | --- | --- | --- | --- | --- | --- | --- | --- | --- |
|  | **30 DAS** | **60 DAS** | **90 DAS** | | **At harvest** | **30 DAS** | **60 DAS** | **90 DAS** | **At harvest** |
| **Factor I: Sowing windows** | | | | | |  |  |  |  |
| W_1_: 1^st^ week of October | 26.25^a^ | 66.0^a^ | 77.78^a^ | | 42.43^a^ | 2.19^a^ | 5.50^a^ | 6.48^a^ | 3.54^a^ |
| W_2_: 2^nd^ week of October | 25.13^a^ | 63.19^a^ | 72.03^ab^ | | 41.52^a^ | 2.09^a^ | 5.27^a^ | 6.01^ab^ | 3.46^a^ |
| W_3_: 3^rd^ week of October | 21.75^b^ | 61.04^ab^ | 65.89^bc^ | | 34.94^b^ | 1.81^b^ | 5.09^ab^ | 5.49^bc^ | 2.91^b^ |
| W_4_: 4^th^ week of October | 18.80^c^ | 55.82^bc^ | 61.51^cd^ | | 31.40^c^ | 1.57^c^ | 4.65^bc^ | 5.13^cd^ | 2.62^c^ |
| W_5_: 1^st^ week of November | 15.33^d^ | 52.86^c^ | 57.26^d^ | | 28.7^c^ | 1.28^d^ | 4.41^c^ | 4.77^d^ | 2.39^c^ |
| **S.Em ±** | **0.65** | **1.66** | **2.19** | | **0.80** | **0.05** | **0.14** | **0.18** | **0.07** |
| **Factor II: Fertility levels** | | | | | |  | | | |
| F_1_: 100 % RDF | 19.13^b^ | 55.61^b^ | 61.74^b^ | | 32.19^c^ | 1.59^b^ | 4.63^b^ | 5.14^b^ | 2.68^c^ |
| F_2_: 150% RDF | 20.83^b^ | 59.41^b^ | 66.76^ab^ | | 35.68^b^ | 1.73^b^ | 4.95^b^ | 5.56^b^ | 2.97^b^ |
| F_3_: 200% RDF | 24.37^a^ | 64.31^a^ | 71.16^a^ | | 39.52^a^ | 2.03^a^ | 5.35^a^ | 6.01^a^ | 3.29^a^ |
| **S.Em ±** | **0.84** | **2.14** | **2.83** | | **1.03** | **0.07** | **0.18** | **0.24** | **0.09** |
| **Interaction (W×F)** | | | | | |  | | | |
| W_1_F_1_: 1^st^ week of Oct + 100 % RDF | 23.18^cd^ | 61.85^a-c^ | 71.85^a-c^ | | 39.01^bc^ | 1.93^cd^ | 5.15^a-c^ | 5.98^a-c^ | 3.25^bc^ |
| W_1_F_2_: 1^st^ week of Oct + 150 % RDF | 25.85^a-c^ | 65.59^ab^ | 77.51^ab^ | | 42.30^ab^ | 2.15^a-c^ | 5.46^ab^ | 6.45^ab^ | 3.52^ab^ |
| W_1_F_3_: 1^st^ week of Oct + 200 % RDF | 29.70^a^ | 70.57^a^ | 83.99^a^ | | 45.97^a^ | 2.47^a^ | 5.88^a^ | 6.99^a^ | 3.83^a^ |
| W_2_F_1_: 2^nd^ week of Oct + 100 % RDF | 23.16^cd^ | 59.11^a-d^ | 66.44^b-d^ | | 38.41^bc^ | 1.93^cd^ | 4.92^a-d^ | 5.53^b-d^ | 3.21^bc^ |
| W_2_F_2_: 2^nd^ week of Oct + 150% RDF | 24.03^bc^ | 62.33^a-c^ | 72.08^a-c^ | | 40.26^bc^ | 2.01^bc^ | 5.19^a-c^ | 6.00^a-c^ | 3.35^bc^ |
| W_2_F_3_: 2^nd^ week of Oct + 200 % RDF | 28.18^ab^ | 68.11^ab^ | 77.55^ab^ | | 45.94^a^ | 2.34^ab^ | 5.67^ab^ | 6.46^ab^ | 3.82^a^ |
| W_3_F_1_: 3^rd^ week of Oct + 100 % RDF | 18.77^d-f^ | 57.07^b-d^ | 60.97^b-d^ | | 30.61^d-f^ | 1.56^d-f^ | 4.75^b-d^ | 5.08^b-d^ | 2.55^d-f^ |
| W_3_F_2_: 3^rd^ week of Oct + 150 % RDF | 21.15^c-e^ | 61.32^a-c^ | 65.62^b-d^ | | 35.98^cd^ | 1.76^c-e^ | 5.11^a-c^ | 5.46^b-d^ | 2.99^cd^ |
| W_3_F_3_: 3^rd^ week of Oct + 200 % RDF | 25.33^a-c^ | 64.71^ab^ | 71.06^a-c^ | | 38.21^bc^ | 2.11^a-c^ | 5.39^ab^ | 5.92^a-c^ | 3.18^bc^ |
| W_4_F_1_: 4^th^ week of Oct + 100 % RDF | 16.71^e-g^ | 51.74^cd^ | 57.06^cd^ | | 27.23^ef^ | 1.39^e-g^ | 4.31^cd^ | 4.75^cd^ | 2.26^ef^ |
| W_4_F_2_: 4^th^ week of Oct + 150 % RDF | 18.35^e-g^ | 55.71^b-d^ | 61.35^b-d^ | | 31.76^de^ | 1.52^e-g^ | 4.62^-d^ | 5.11^b-d^ | 2.64^de^ |
| W_4_F_3_: 4^th^ week of Oct + 200 % RDF | 21.33^c-e^ | 60.01^a-d^ | 66.11^b-d^ | | 35.28^cd^ | 1.77^c-e^ | 5.01^a-d^ | 5.50^b-d^ | 2.94^cd^ |
| W_5_F_1_: 1^st^ week of Nov + 100 % RDF | 13.86^g^ | 48.29^d^ | 52.41^d^ | | 25.73^f^ | 1.15^g^ | 4.02^d^ | 4.36^d^ | 2.14^f^ |
| W_5_F_2_: 1^st^ week of Nov + 150 % RDF | 14.79^fg^ | 52.11^cd^ | 57.27^cd^ | | 28.21^ef^ | 1.23^fg^ | 4.34^cd^ | 4.77^cd^ | 2.35^ef^ |
| W_5_F_3_: 1^st^ week of Nov + 200 % RDF | 17.34^e-g^ | 58.16^a-d^ | 62.08^b-d^ | | 32.19^de^ | 1.44^e-g^ | 4.84^b-d^ | 5.17^b-d^ | 2.68^de^ |
| **S.Em ±** | **1.45** | **3.71** | **4.90** | | **1.78** | **0.12** | **0.30** | **0.40** | **0.15** |
| C_1_: Control | 8.1 | 23.81 | 28.9 | 8.6 | | 0.7 | 2.0 | 2.4 | 0.67 |
| **S.Em ±** | **1.41** | **3.94** | **4.78** | **1.75** | | **0.11** | **0.32** | **0.39** | **0.14** |

**S5 Table. Total dry matter production per plant at different growth stages of *rabi* maize as influenced by sowing windows and fertility levels**

| **Treatment** | **Total dry matter production (g plant^-1^)** | | | |
| --- | --- | --- | --- | --- |
|  | **30 DAS** | **60 DAS** | **90 DAS** | **At harvest** |
| **Factor I: Sowing windows** | | | | |
| W_1_: 1^st^ week of October | 13.51^a^ | 104.53^a^ | 229.74^a^ | 280.13^a^ |
| W_2_: 2^nd^ week of October | 12.98^a^ | 101.84^a^ | 222.27^a^ | 273.91^a^ |
| W_3_: 3^rd^ week of October | 11.29^b^ | 91.83^b^ | 203.73^b^ | 252.78^b^ |
| W_4_: 4^th^ week of October | 11.13^b^ | 82.44^c^ | 194.38^bc^ | 243.15^bc^ |
| W_5_: 1^st^ week of November | 9.79^c^ | 77.29^c^ | 184.20^c^ | 231.74^c^ |
| **S.Em ±** | **0.28** | **2.29** | **4.67** | **3.97** |
| **Factor II: Fertility levels** | | | | |
| F_1_: 100 % RDF | 11.14^b^ | 85.03^b^ | 194.87^b^ | 247.02^b^ |
| F_2_: 150% RDF | 11.68^ab^ | 90.86^b^ | 206.71^ab^ | 256.42^ab^ |
| F_3_: 200% RDF | 12.41^a^ | 98.86^a^ | 219.01^a^ | 265.57^a^ |
| **S.Em ±** | **0.36** | **2.96** | **6.02** | **5.13** |
| **Interaction (W×F)** | | | | |
| W_1_F_1_: 1^st^ week of Oct + 100 % RDF | 12.72^a-d^ | 97.83^a-f^ | 216.63^a-e^ | 271.15^a-d^ |
| W_1_F_2_: 1^st^ week of Oct + 150 % RDF | 13.61^ab^ | 103.63^a-c^ | 229.23^a-c^ | 278.03^a-c^ |
| W_1_F_3_: 1^st^ week of Oct + 200 % RDF | 14.23^a^ | 112.16^a^ | 243.36^a^ | 291.19^a^ |
| W_2_F_1_: 2^nd^ week of Oct + 100 % RDF | 12.26^a-e^ | 94.12^b-f^ | 209.24^a-f^ | 265.36^a-e^ |
| W_2_F_2_: 2^nd^ week of Oct + 150% RDF | 12.93^a-c^ | 101.56^a-d^ | 224.48^a-d^ | 273.71^a-c^ |
| W_2_F_3_: 2^nd^ week of Oct + 200 % RDF | 13.73^ab^ | 110.0^ab^ | 235.16^ab^ | 282.66^ab^ |
| W_3_F_1_: 3^rd^ week of Oct + 100 % RDF | 10.85^d-g^ | 85.63^d-g^ | 191.56^d-f^ | 242.36^d-g^ |
| W_3_F_2_: 3^rd^ week of Oct + 150 % RDF | 11.26^c-g^ | 91.42^c-g^ | 204.43^b-f^ | 255.38^b-f^ |
| W_3_F_3_: 3^rd^ week of Oct + 200 % RDF | 11.76^b-f^ | 98.56^a-e^ | 215.66^a-d^ | 260.61^b-f^ |
| W_4_F_1_: 4^th^ week of Oct + 100 % RDF | 10.61^e-g^ | 76.63^gh^ | 182.66^ef^ | 234.56^fg^ |
| W_4_F_2_: 4^th^ week of Oct + 150 % RDF | 10.83^d-g^ | 81.20^f-h^ | 194.32^c-f^ | 242.93^d-g^ |
| W_4_F_3_: 4^th^ week of Oct + 200 % RDF | 12.04^b-e^ | 89.52^c-g^ | 206.13^b-f^ | 251.95^c-f^ |
| W_5_F_1_: 1^st^ week of Nov + 100 % RDF | 9.31^g^ | 71.16^h^ | 174.32^f^ | 221.66^g^ |
| W_5_F_2_: 1^st^ week of Nov + 150 % RDF | 9.85^fg^ | 76.52^gh^ | 183.06^ef^ | 232.09^fg^ |
| W_5_F_3_: 1^st^ week of Nov + 200 % RDF | 10.26^e-g^ | 84.21^e-h^ | 194.81^c-f^ | 241.45^e-g^ |
| **S.Em ±** | **0.62** | **5.13** | **10.43** | **8.88** |
| C_1_: Control | 6.4 | 61.72 | 136.71 | 174.6 |
| **S.Em ±** | **0.64** | **4.99** | **10.09** | **8.66** |

**S6 Table. Absolute growth rate and crop growth rate at different growth stages of *rabi* maize as influenced by sowing windows and fertility levels**

| **Treatment** | **Absolute growth rate  (g day^-1^)** | | | **Crop growth rate  (g m^-2^ day^-1^)** | | |
| --- | --- | --- | --- | --- | --- | --- |
|  | **30-60 DAS** | **60-90 DAS** | **90 DAS-Harvest** | **30-60 DAS** | **60-90 DAS** | **90 DAS- Harvest** |
| **Factor I: Sowing windows** | | | | | | |
| W_1_: 1^st^ week of October | 3.03^a^ | 4.17^a^ | 1.72^a^ | 25.28^a^ | 34.78^a^ | 14.35^a^ |
| W_2_: 2^nd^ week of October | 2.96^ab^ | 4.01^ab^ | 1.68^a^ | 24.69^ab^ | 33.45^a^ | 14.00^a^ |
| W_3_: 3^rd^ week of October | 2.68^b^ | 3.73^ab^ | 1.63^a^ | 22.37^b^ | 31.08^a^ | 13.62^a^ |
| W_4_: 4^th^ week of October | 2.38^c^ | 3.73^ab^ | 1.62^a^ | 19.81^c^ | 31.09^a^ | 13.55^a^ |
| W_5_: 1^st^ week of November | 2.25^c^ | 3.56^b^ | 1.47^a^ | 18.75^c^ | 29.70^a^ | 13.23^a^ |
| **S.Em ±** | **0.08** | **0.13** | **0.08** | **0.63** | **1.42** | **1.65** |
| **Factor II: Fertility levels** | | | | | | |
| F_1_: 100 % RDF | 2.46^b^ | 3.66^a^ | 1.48^a^ | 20.53^b^ | 30.51^a^ | 12.93^a^ |
| F_2_: 150% RDF | 2.64^b^ | 3.86^a^ | 1.63^a^ | 21.99^b^ | 32.18^a^ | 13.71^a^ |
| F_3_: 200% RDF | 2.88^a^ | 4.00^a^ | 1.75^a^ | 24.02^a^ | 33.37^a^ | 14.60^a^ |
| **S.Em ±** | **0.10** | **0.17** | **0.10** | **0.81** | **1.83** | **2.12** |
| **Interaction (W×F)** | | | | | | |
| W_1_F_1_: 1^st^ week of Oct + 100 % RDF | 2.84^a-d^ | 3.96^a^ | 1.58^ab^ | 23.64^c-e^ | 33.01^a^ | 13.21^a^ |
| W_1_F_2_: 1^st^ week of Oct + 150 % RDF | 3.00^a-c^ | 4.18^a^ | 1.70^ab^ | 25.01^b-e^ | 34.89^a^ | 14.23^a^ |
| W_1_F_3_: 1^st^ week of Oct + 200 % RDF | 3.26^a^ | 4.37^a^ | 1.86^a^ | 27.20^a-c^ | 36.44^a^ | 15.59^a^ |
| W_2_F_1_: 2^nd^ week of Oct + 100 % RDF | 2.72^a-e^ | 3.84^a^ | 1.59^ab^ | 22.70^c-f^ | 32.01^a^ | 13.28^a^ |
| W_2_F_2_: 2^nd^ week of Oct + 150% RDF | 2.95^a-c^ | 4.03^a^ | 1.62^ab^ | 24.62^b-e^ | 33.58^a^ | 13.55^a^ |
| W_2_F_3_: 2^nd^ week of Oct + 200 % RDF | 3.21^ab^ | 4.17^a^ | 1.81^a^ | 26.73^a-d^ | 34.76^a^ | 15.34^a^ |
| W_3_F_1_: 3^rd^ week of Oct + 100 % RDF | 2.49^c-f^ | 3.52^a^ | 1.49^ab^ | 20.76^e-g^ | 29.31^a^ | 12.48^a^ |
| W_3_F_2_: 3^rd^ week of Oct + 150 % RDF | 2.67^b-e^ | 3.77^a^ | 1.69^ab^ | 22.26^d-f^ | 31.40^a^ | 14.14^a^ |
| W_3_F_3_: 3^rd^ week of Oct + 200 % RDF | 2.89^a-d^ | 3.91^a^ | 1.70^ab^ | 24.09^b-e^ | 32.55^a^ | 14.24^a^ |
| W_4_F_1_: 4^th^ week of Oct + 100 % RDF | 2.20^ef^ | 3.53^a^ | 1.52^ab^ | 28.34^ab^ | 29.45^a^ | 12.72^a^ |
| W_4_F_2_: 4^th^ week of Oct + 150 % RDF | 2.35^d-f^ | 3.77^a^ | 1.60^ab^ | 29.56^a^ | 31.43^a^ | 13.50^a^ |
| W_4_F_3_: 4^th^ week of Oct + 200 % RDF | 2.58^c-f^ | 3.89^a^ | 1.72^ab^ | 21.53^e-g^ | 32.40^a^ | 14.43^a^ |
| W_5_F_1_: 1^st^ week of Nov + 100 % RDF | 2.06^f^ | 3.45^a^ | 1.23^b^ | 17.18^g^ | 28.77^a^ | 12.96^a^ |
| W_5_F_2_: 1^st^ week of Nov + 150 % RDF | 2.22^ef^ | 3.55^a^ | 1.55^ab^ | 18.53^fg^ | 29.60^a^ | 13.12^a^ |
| W_5_F_3_: 1^st^ week of Nov + 200 % RDF | 2.46^c-f^ | 3.69^a^ | 1.63^ab^ | 20.54^e-g^ | 30.72^a^ | 13.61^a^ |
| **S.Em ±** | **0.17** | **0.29** | **0.17** | **1.40** | **3.18** | **3.68** |
| C_1_: Control | 1.8 | 2.5 | 1.3 | 15.4 | 20.8 | 10.5 |
| **S.Em ±** | **0.16** | **0.28** | **0.16** | **1.36** | **3.07** | **3.57** |

**S7 Table. Accumulated growing degree days at different growth stages of *rabi* maize as influenced by sowing windows and fertility levels**

| **Treatment** | **Growing degree days (^0^ C day)** | | | |
| --- | --- | --- | --- | --- |
|  | **Emergence** | **Tasselling** | **Silking** | **Physiological maturity** |
| **Factor I: Sowing windows** | | | | |
| W_1_: 1^st^ week of October | 78.4^d^ | 835.0^a^ | 921.4^a^ | 1530.1^a^ |
| W_2_: 2^nd^ week of October | 87.6^b^ | 806.0^b^ | 883.3^b^ | 1493.4^a^ |
| W_3_: 3^rd^ week of October | 83.4^c^ | 762.5^c^ | 850.2^c^ | 1466.9^b^ |
| W_4_: 4^th^ week of October | 88.1^b^ | 746.1^d^ | 829.0^d^ | 1444.9^c^ |
| W_5_: 1^st^ week of November | 89.9^a^ | 569.6^e^ | 648.8^e^ | 1242.4^d^ |
| **S.Em ±** | **0.23** | **1.51** | **1.94** | **2.53** |
| **Factor II: Fertility levels** | | | | |
| F_1_: 100 % RDF | 84.9^b^ | 726.1^c^ | 799.5^c^ | 1392.7^c^ |
| F_2_: 150% RDF | 84.9^b^ | 744.3^b^ | 825.5^b^ | 1421.0^b^ |
| F_3_: 200% RDF | 86.6^a^ | 761.1^a^ | 854.6^a^ | 1493.5^a^ |
| **S.Em ±** | **0.30** | **1.95** | **2.50** | **3.27** |
| **Interaction (W×F)** | | | | |
| W_1_F_1_: 1^st^ week of Oct + 100 % RDF | 74.6^h^ | 810.6^d^ | 880.2^c^ | 1471.4^e^ |
| W_1_F_2_: 1^st^ week of Oct + 150 % RDF | 79.5^g^ | 839.8^b^ | 923.0^b^ | 1527.8^c^ |
| W_1_F_3_: 1^st^ week of Oct + 200 % RDF | 81.0^fg^ | 854.5^a^ | 960.8^a^ | 1591.1^a^ |
| W_2_F_1_: 2^nd^ week of Oct + 100 % RDF | 87.1^cd^ | 784.1^e^ | 859.0^d^ | 1444.4^f^ |
| W_2_F_2_: 2^nd^ week of Oct + 150% RDF | 87.1^cd^ | 809.4^d^ | 872.3^c^ | 1474.4^e^ |
| W_2_F_3_: 2^nd^ week of Oct + 200 % RDF | 88.6^bc^ | 824.6^c^ | 918.7^b^ | 1561.3^b^ |
| W_3_F_1_: 3^rd^ week of Oct + 100 % RDF | 81.5^f^ | 739.7^hi^ | 821.2^f^ | 1433.4^f^ |
| W_3_F_2_: 3^rd^ week of Oct + 150 % RDF | 84.4^e^ | 760.7^f^ | 846.8^de^ | 1445.0^f^ |
| W_3_F_3_: 3^rd^ week of Oct + 200 % RDF | 84.4^e^ | 787.2^e^ | 882.6^c^ | 1522.4^c^ |
| W_4_F_1_: 4^th^ week of Oct + 100 % RDF | 93.2^a^ | 734.7^i^ | 808.4^g^ | 1397.8^g^ |
| W_4_F_2_: 4^th^ week of Oct + 150 % RDF | 84.1^e^ | 747.2^gh^ | 840.4^e^ | 1440.0^f^ |
| W_4_F_3_: 4^th^ week of Oct + 200 % RDF | 86.9^d^ | 756.4^fg^ | 838.0^e^ | 1497.0^d^ |
| W_5_F_1_: 1^st^ week of Nov + 100 % RDF | 87.9^b-d^ | 561.6^k^ | 628.7^j^ | 1213.9^i^ |
| W_5_F_2_: 1^st^ week of Nov + 150 % RDF | 89.4^b^ | 564.6^k^ | 644.8^i^ | 1218.0^i^ |
| W_5_F_3_: 1^st^ week of Nov + 200 % RDF | 92.3^a^ | 582.7^j^ | 672.7^h^ | 1295.5^h^ |
| **S.Em ±** | **0.51** | **3.36** | **4.33** | **6.67** |
| C_1_: Control | 81.0 | 689.4 | 767.4 | 1363.2 |
| **S.Em ±** | **0.50** | **3.27** | **4.43** | **6.64** |
